# Supplementary material for: Utilization of a Wheat50K SNP Microarray-Derived High-Density Genetic Map for QTL Mapping of Plant Height and Grain Traits in Wheat
Source: Plants (Basel). 2021 Jun 8;10(6):1167. doi: 10.3390/plants10061167 (PMC8229693; doi:10.3390/plants10061167)
Supplement: Supplementary file 1 [file plants-10-01167-s001.zip › sup/Supplementary Figure 1 Correlation among plant height, TGW, grain length and grain width in different environments..pdf]

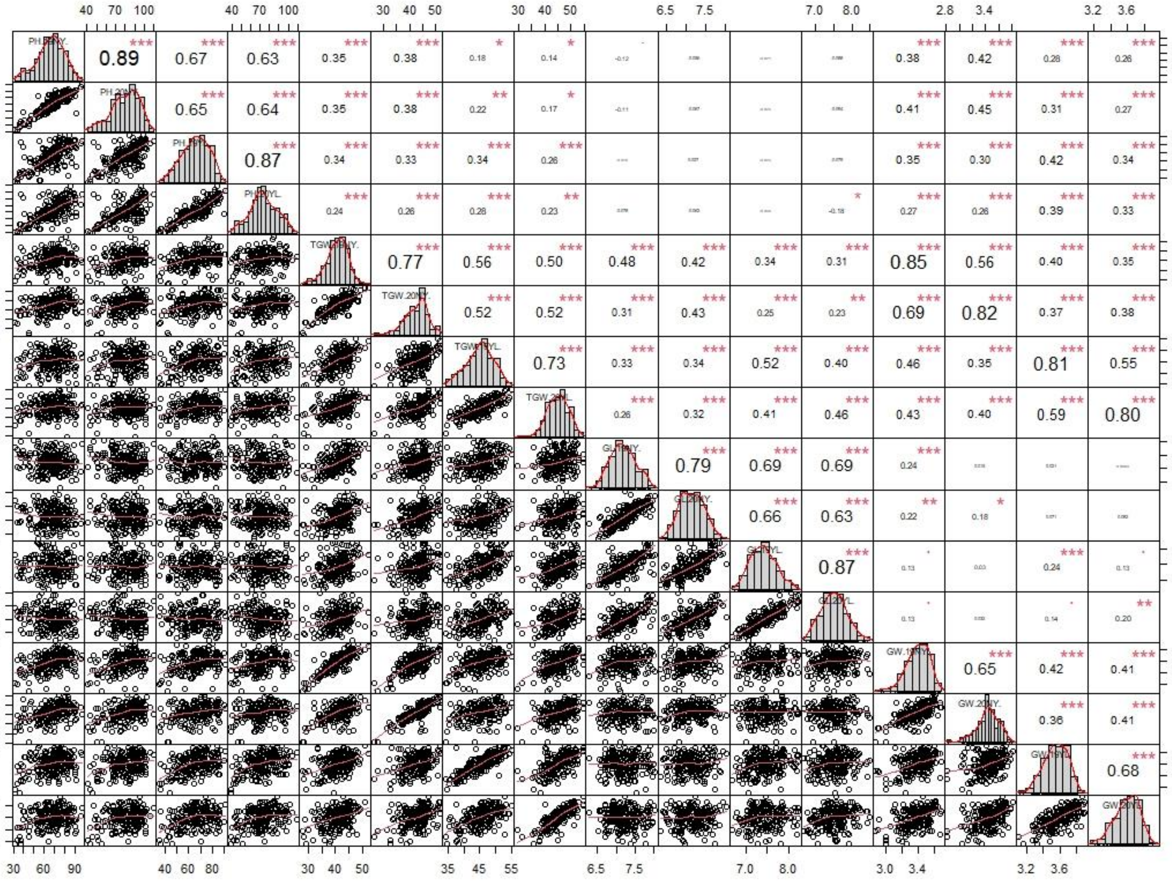

Supplementary Figure 1. Correlation among plant height, TGW, grain length and grain width in different environments.

Note: Horizontally and vertically, each four represents a phenotype, and each small cell represents a different environment.
