# Supplementary material for: Utilization of a Wheat50K SNP Microarray-Derived High-Density Genetic Map for QTL Mapping of Plant Height and Grain Traits in Wheat
Source: Plants (Basel). 2021 Jun 8;10(6):1167. doi: 10.3390/plants10061167 (PMC8229693; doi:10.3390/plants10061167)
Supplement: Supplementary file 1 [file plants-10-01167-s001.zip › sup/Supplementary Figure 2 Based on the 660K chip labeling, the SNP markers that differ between the two parents are detected.pdf]

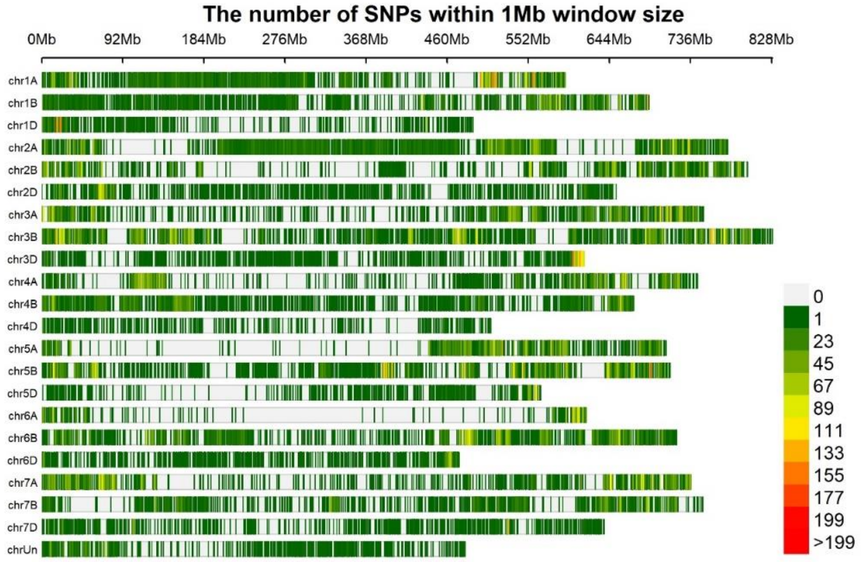

Supplementary Figure 2. Based on the 660K chip labeling, the SNP markers that differ between the two parents are detected.

Note: The largest gap appears on chromosomes 5A and 6A, the gaps are 65.17015MB and 135.1196MB, respectively.
