# Supplementary material for: Utilization of a Wheat50K SNP Microarray-Derived High-Density Genetic Map for QTL Mapping of Plant Height and Grain Traits in Wheat
Source: Plants (Basel). 2021 Jun 8;10(6):1167. doi: 10.3390/plants10061167 (PMC8229693; doi:10.3390/plants10061167)
Supplement: Supplementary file 1 [file plants-10-01167-s001.zip › sup/Supplementary Figure 3 x-sec-p1x-sec-p2 marker genotyping results..pdf]

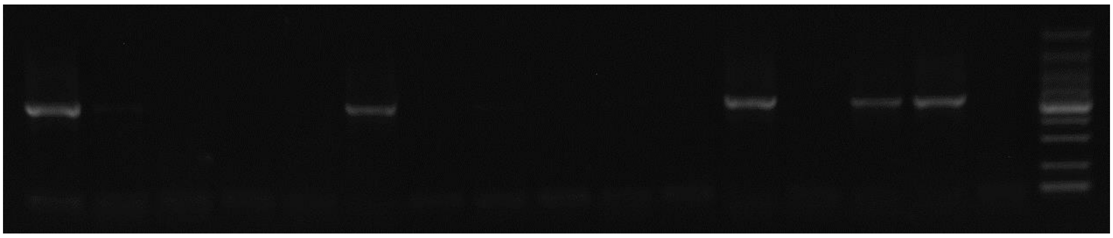

Supplementary Figure 3. x-sec-p1/x-sec-p2 marker genotyping results.

Note: From left to right: L76, L77, L79, L80, L81, L82, L83, L84, L85, L86, L87 L88, L89, L90, Xinong1376, Xiaoyan81**.**
