# Supplementary material for: Utilization of a Wheat50K SNP Microarray-Derived High-Density Genetic Map for QTL Mapping of Plant Height and Grain Traits in Wheat
Source: Plants (Basel). 2021 Jun 8;10(6):1167. doi: 10.3390/plants10061167 (PMC8229693; doi:10.3390/plants10061167)
Supplement: Supplementary file 1 [file plants-10-01167-s001.zip › sup/Supplementary Figure 4 QTL mapping of plant height and TGW phenotypes at genome-wide level.pdf]

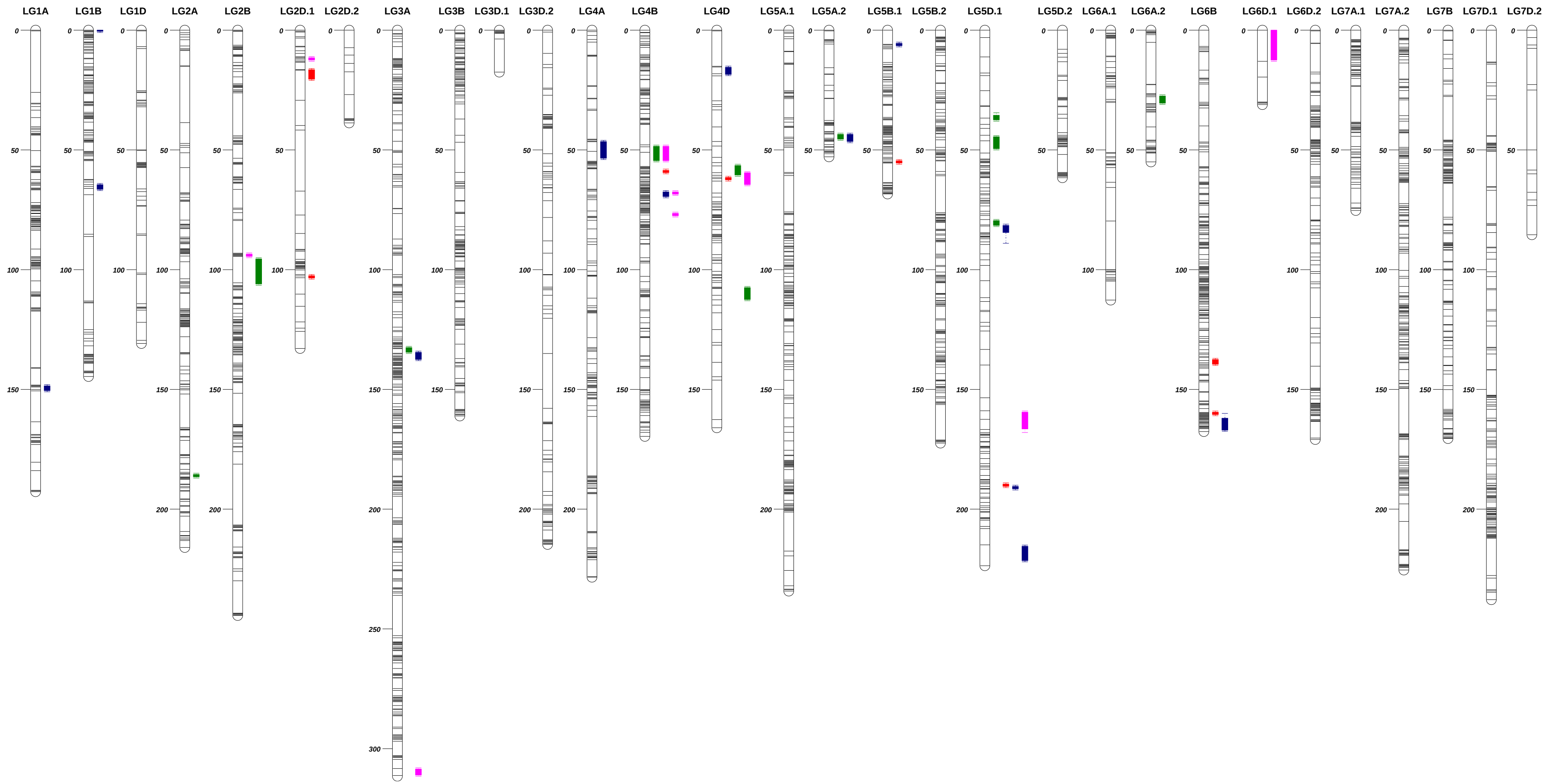

Supplementary Figure 4. QTL mapping of plant height and TGW phenotypes at genome-wide level. The right block of the genetic map is the QTL site, and red is regarded as the QTL site of plant height, green as the QTL site of 1000-grain weight, blue as the QTL site of grain length, and pink as the QTL site of grain width.
