# Supplementary material for: Utilization of a Wheat50K SNP Microarray-Derived High-Density Genetic Map for QTL Mapping of Plant Height and Grain Traits in Wheat
Source: Plants (Basel). 2021 Jun 8;10(6):1167. doi: 10.3390/plants10061167 (PMC8229693; doi:10.3390/plants10061167)
Supplement: Supplementary file 1 [file plants-10-01167-s001.zip › sup/Supplementary Figure 5 KASP_AX-109316968 and KASP_AX-109333198 markers genotyping results..pdf]

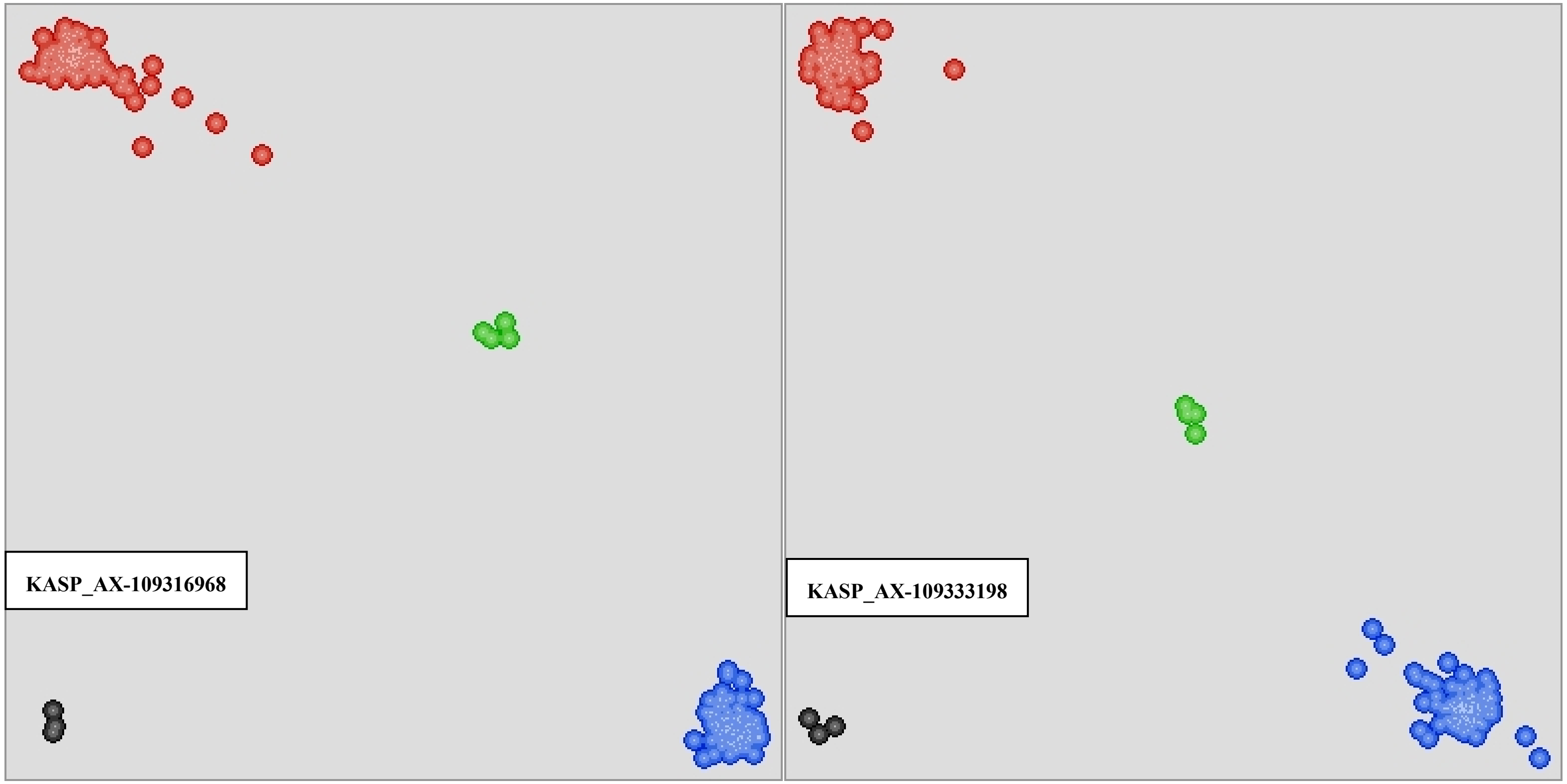

Supplementary Figure 5. KASP_AX-109316968 and KASP_AX-109333198 markers genotyping results.

Note:KASP_AX-109316968, blue represents the allele of Xinong1376, red represents the allele of Xiaoyan81, and green represents the heterozygous genotype.KASP_AX-109333198, blue represents the allele of Xiaoyan81, red represents the allele of Xinong1376, and green represents the heterozygous genotype.
