# Supplementary material for: Utilization of a Wheat50K SNP Microarray-Derived High-Density Genetic Map for QTL Mapping of Plant Height and Grain Traits in Wheat
Source: Plants (Basel). 2021 Jun 8;10(6):1167. doi: 10.3390/plants10061167 (PMC8229693; doi:10.3390/plants10061167)
Supplement: Supplementary file 1 [file plants-10-01167-s001.zip › sup/Supplementary Figure 6 The pedigree of Xinong 1376 and Xiaoyan 81.pdf]

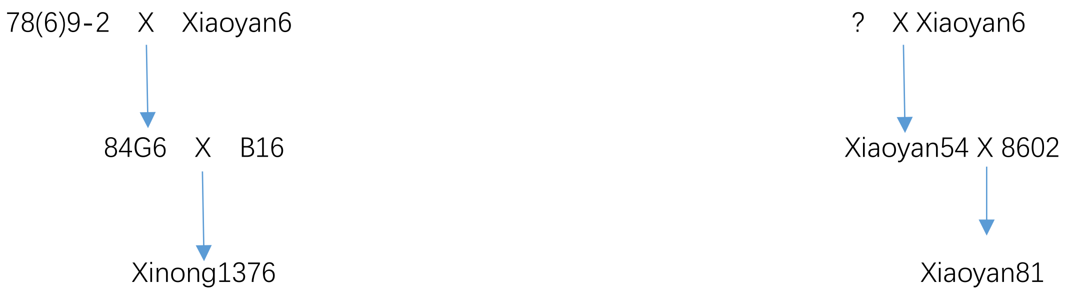

Supplementary Figure 6. The pedigree of Xinong 1376 and Xiaoyan 81

Note: Xinong 1376 is a large-spike weak spring variety. Xiaoyan 81 is a multi-spike semi-winter variety.
